# Are semi-automated software program designed for adults accurate for the identification of vertebral fractures in children?

**DOI:** 10.1007/s00330-019-06250-4

**Published:** 2019-05-22

**Authors:** Fawaz F. Alqahtani, Fabrizio Messina, Amaka C. Offiah

**Affiliations:** 1 Academic Unit of Child Health, Department of Oncology and Metabolism, University of Sheffield, Medical School, University of Sheffield, Street Building, Western Bank, Sheffield, S10 2TH UK; 2 Department of Radiological Sciences, College of Applied Medical Sciences, Najran University, Najran, Kingdom of Saudi Arabia; 3 School of Health and Related Research, University of Sheffield, Sheffield, UK; 4 Radiology Department, Sheffield Children’s NHS Foundation Trust, Sheffield, UK

**Keywords:** Osteoporosis, Children, DXA scan, Vertebral, Paediatric

## Abstract

**Objectives:**

To assess whether diagnostic accuracy of morphometric vertebral fracture (VF) diagnosis in children can be improved using AVERT™ (a 33-point semi-automated program developed for VF diagnosis in adults) compared with SpineAnalyzer™ (a 6-point program), which has previously been shown to be of insufficient accuracy.

**Materials and methods:**

Lateral spine radiographs (XR) and dual-energy X-ray absorptiometry (DXA) scans of 50 children and young people were analysed by two observers using two different programs (AVERT™ and SpineAnalyzer™). Diagnostic accuracy (sensitivity, specificity, false-negative (FN) and false-positive rates (FP)) was calculated by comparing with a previously established consensus arrived at by three experienced paediatric musculoskeletal radiologists, using a simplified algorithm-based qualitative scoring system. Observer agreement was calculated using Cohen’s kappa.

**Results:**

For XR, overall sensitivity, specificity, FP and FN rates using AVERT™ were 36%, 95%, 5% and 64% respectively and 26%, 98%, 2% and 75% respectively, using SpineAnalyzer™. For DXA, overall sensitivity, specificity, FP and FN rates using AVERT™ were 41%, 91%, 9% and 59% respectively and 31%, 96%, 4% and 69% respectively, using SpineAnalyzer. Reliability (kappa) ranged from 0.34 to 0.37 (95%CI, 0.26–0.46) for AVERT™ and from 0.26 to 0.31 (95%CI, 0.16–0.44) for SpineAnalyzer™. Inter- and intra-observer agreement ranged from 0.41 to 0.47 for AVERT™ and from 0.50 to 0.79 for SpineAnalyzer™.

**Conclusion:**

AVERT™ has slightly higher accuracy but lower observer reliability for the representation of vertebral morphometry in children when compared with SpineAnalyzer™. However, neither software program is satisfactorily reliable for VF diagnosis in children.

**Key Points:**

*• SpineAnalyzer™ and AVERT™ have low diagnostic accuracy and observer agreement when compared to three paediatric radiologists’ readings for the diagnosis of vertebral fractures (VF) in children.*

*• Neither AVERT™ nor SpineAnalyzer™ is satisfactorily reliable for VF diagnosis in children.*

*• Development of specific paediatric software and normative values (incorporating age-related physiological variation in children) is required.*

## Introduction

Low bone mass is characterised by structural deterioration of bone tissue, leading to bone fragility and increased susceptibility to fractures, especially of the spine and long bones. According to the International Society for Clinical Densitometry (ISCD), one or multiple vertebral fractures (VF)—identified by a 20% reduction in vertebral body height—indicates bone fragility, in the absence of local disease or significant trauma [1].

Osteoporotic VFs are increasingly recognised in children as a vital sign of low bone mineral density (BMD) whether primary, e.g. osteogenesis imperfecta [2], or secondary, e.g. acute lymphoblastic leukaemia, rheumatological conditions, Duchenne muscular dystrophy and glucocorticoid use [1, 3]. Moreover, children who have been identified with VFs, especially those with osteogenesis imperfecta and Duchenne muscular dystrophy, are more likely to have multiple VFs [4, 5]. Early radiological diagnosis and accurate identification of patients with prevalent VF are important for the effective targeting of therapy to prevent new fractures.

Currently, the gold standard for identifying VFs in children is the lateral spine radiograph. Recent studies have shown that spine images acquired by dual-energy X-ray absorptiometry (DXA) are comparable to radiographs [6–8], allowing reduced exposure to radiation. The diagnosis of VFs from DXA is termed vertebral fracture assessment (VFA).

There is no standardised technique for objective diagnosis of VFs in children, and clinical studies have shown that there is significant inter- and intra-observer variability in this population [3, 9–11]. Moreover, the limited studies carried out to assess morphometric analysis (MXA) using a 6-point semi-automated software program in children have also shown poor observer reliability [8, 12].

The aim of this study, therefore, was to assess whether observer reliability and diagnostic accuracy of MXA for the identification of VF in children would be improved by using a 33-point semi-automated program compared with the 6-point program.

## Materials and methods

### Study population

The study population included 100 (50 DXA-VFA and 50 radiographic (XR)) lateral spine images that were obtained as part of a larger prospective study involving 137 children; these children were recruited between November 2011 and February 2014 [6, 12]. The sample selection was randomly made using a random number generator. All images belonged to patients recruited from a single centre. All DXA and XR were performed on the same day, with patients in the lateral decubitus position for both studies [6]. The majority of patients (80%) were those with suspected reduced BMD, e.g. osteogenesis imperfecta, inflammatory bowel disease, rheumatological conditions, and cystic fibrosis, attending the metabolic bone clinic for iDXA and lateral spine radiographs. Details of image acquisition have previously been reported [6]. The remaining 20% of patients were those attending spine clinics for suspected scoliosis.

### Ethics statement

For the main study, approval of the Local Research Ethics Committee was sought and obtained, but was not separately required for this study. The study was registered with the local Research and Innovation Department prior to commencement.

### Image analysis

XR and VFA images were independently evaluated for VF by a research radiographer (R1) and an expert paediatric radiologist (R2), using two different semi-automated programs: (1) SpineAnalyzer™ (Optasia Medical) and (2) AVERT™ (Optasia Medical). SpineAnalyzer™ is Optasia’s software based on an active appearance model. AVERT™ is partially derived from SpineAnalyzer™, but uses the latest appearance modelling technology (random forest regression voting constrained local models) from the University of Manchester software libraries. Potentially, therefore, AVERT™ might be expected to provide more accurate fits [13].

Prior to commencing the study, R1 was trained to use the software programs by a research associate in computing science and an expert radiologist (MSK research radiology fellow), learning from non-study spine images. In order to reduce observer bias, XR and VFA images were analysed on different days, in random order without accessing the subject’s clinical information and also blinded to any previous analyses. Repeat scoring was performed on 10 randomly selected patients blinded to previous reads.

**Fig. 1:**
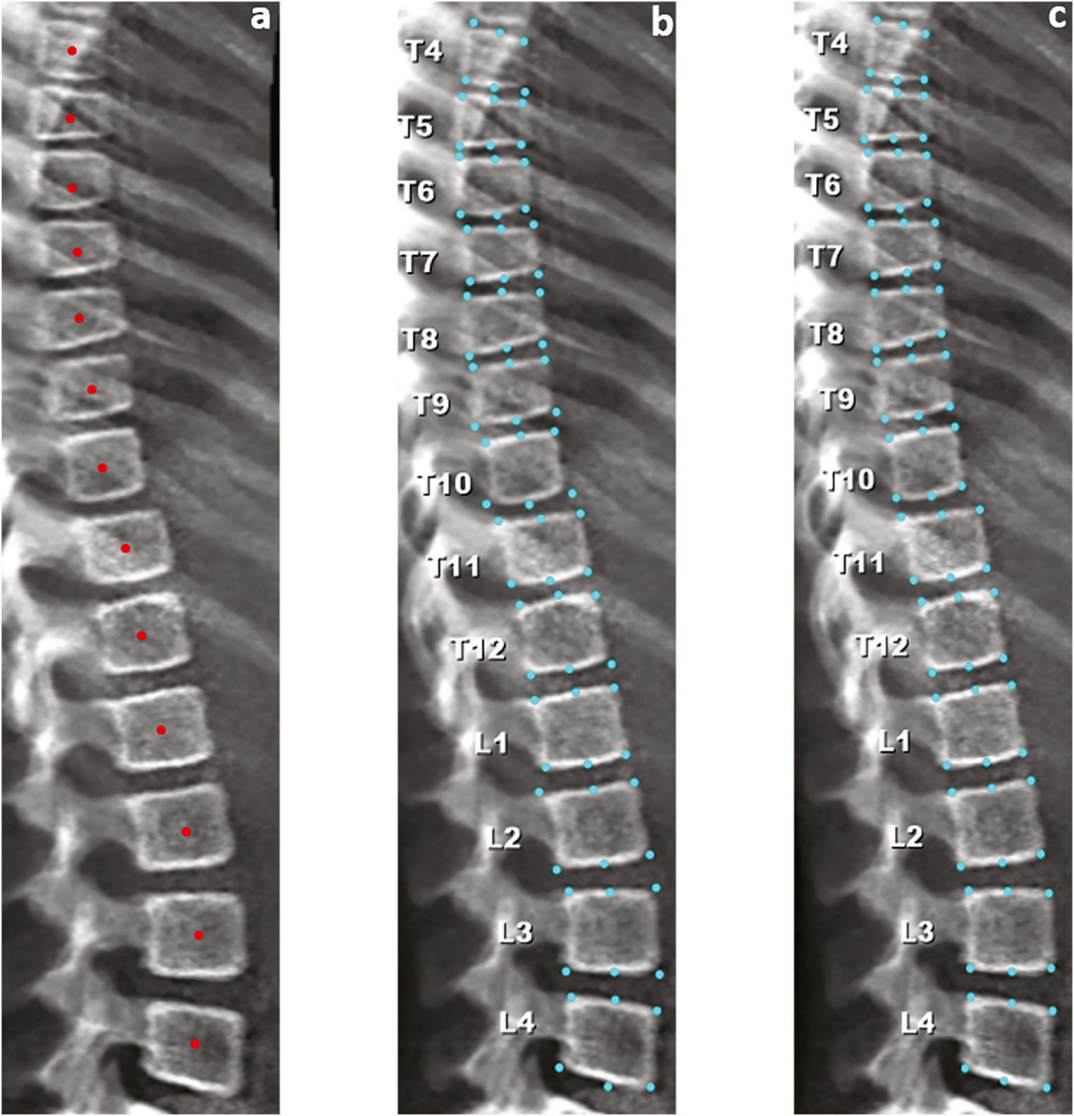
Analysing an iDXA lateral spine image using SpineAnalyzer™. **a** Placement of a single point at the centre of each vertebral body. **b** Automatic 6-point annotation. **c** Manual correction of 6 points (e.g. anterior points of T10 and T12)

In line with the process associated with semi-automated analysis using SpineAnalyzer™, for each individual image (VFA or XR), the observer tracked T4 to L4 vertebral bodies by placing a single point at their centre (Fig. 1a) and indicating to the software the highest identified vertebral body (for example, T4). Subsequently, the program takes cognisance of all the identified vertebral bodies between T4 and L4 and automatically identifies 6 points that correspond to the midpoints of the superior and inferior endplates and the four corners of each vertebral body (Fig. 1b), although these can be modified as necessary (Fig. 1c). Importantly, the software does not recognise vertebral bodies above T4 or below L4, although unreadable vertebral bodies between these levels can be omitted from the readings. Once the 6 points have been placed, anterior, middle and posterior vertebral heights are automatically determined by the software and, with the help of such measurements, the ((anterior: posterior), (middle: posterior), (posterior: posterior^+1^ and posterior: posterior^−1^)) height ratios are calculated (+ 1 and − 1 indicate the vertebrae immediately above [+ 1] and below [− 1] the vertebra of interest). The vertebral bodies are then categorised according to the height loss ratio: height loss of 20–25% (mild), height loss of 25–40% (moderate) or height loss more than 40% (severe), based on the semi-quantitative scoring system developed by Genant et al [14].

**Fig. 2:**
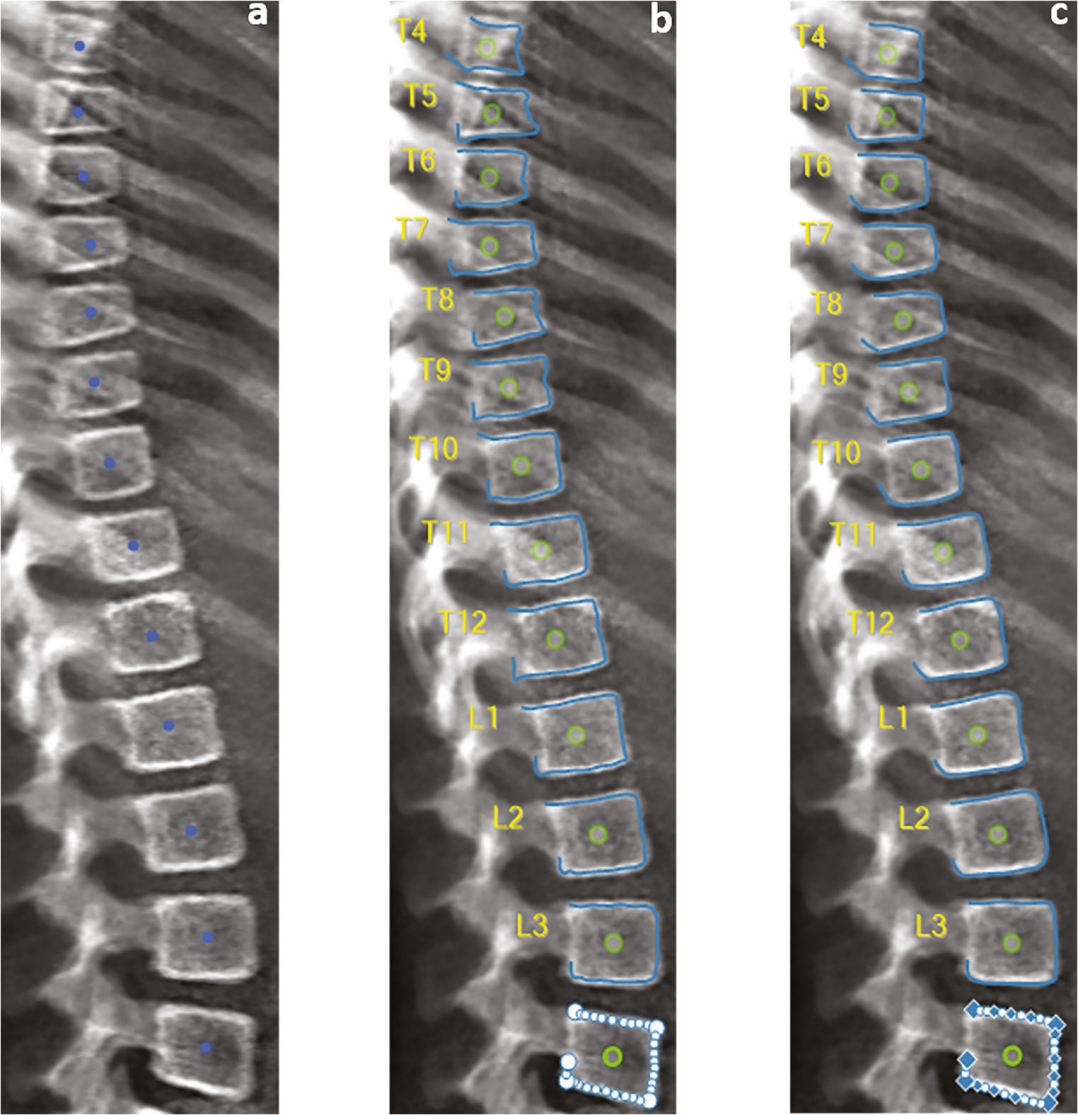
Analysing an iDXA lateral spine image using AVERT™. **a** Placement of a single point at the centre of each vertebral body. **b** Automatic 33-point annotation. **c** Manual correction of 33 points at L4

In the case of AVERT™, all lateral XR and VFA images (T4–L4) were analysed as follows: initial manual targeting of the centres of the vertebral bodies of interest (Fig. 2a), then the software numbers the vertebral bodies accordingly. The software then automatically finds the positions of landmarks to enable a 33-point measurement (Fig. 2b) for each vertebral body: 11 on the upper end-plate, 8 anteriorly, 11 on the lower end-plate, and 3 posteriorly. The software then allows these points to be moved by the observer, if deemed necessary, to correct any fitting failures (Fig. 2c). Subsequently, the confirmed points are used by the software to calculate the anterior, middle and posterior vertebral heights, which are used for the determination of the shape of any deformity. From these measurements, the ((anterior: posterior), (middle: posterior), (posterior: posterior^+2^ and posterior: posterior^−2^)) height ratios are calculated (+2 and − 2 indicate the four neighbouring vertebrae, the two immediately above [+ 2] and the two immediately below [− 2] the vertebra of interest). Thereafter, the vertebral bodies are classified as per their height ratios, on the basis of Genant’s scoring system [14].

For this study, in terms of identifying vertebral levels, the first vertebral body that was not associated with a pair of ribs was marked as L1, with the lowermost vertebral body associated with ribs then marked as T12.

For both programs, the operator is able to move the points for improved fit to vertebral shape. The time to conduct MXA for both programs was measured for R1 and R2 on 20 randomly selected images.

### Statistical analysis

SPSS statistics software version 24 (IBM) and Microsoft® Excel 2016 were employed for data analysis. The reference standard for diagnostic accuracy (sensitivity, specificity, false-positive and false-negative rates) calculations were taken from a previous consensus reached by three paediatric radiologists using a simplified algorithm-based qualitative (sABQ) scoring system [11]. For these calculations of diagnostic accuracy, all sABQ, SpineAnalyzer™ and AVERT™ scores of 0 or 1 were interpreted as, “no clinically significant fracture”. Inter- and intra-observer agreements were calculated using Cohen’s kappa with a 95% confidence interval [CI].

## Results

The mean age of the 50 subjects at the time of image acquisition was 9.6 years (range 5 to 15) and 21 (42%) were male.

According to the reference standard, 34 (68%) had at least one fracture. Amongst these 34 patients, there was a total of 175 VFs, 132 (75%) were mild, 41 (23%) were moderate and 2 (1%) were severe. Only 2 of the 34 patients (4%) had severe fractures.

A total of 2600 individual vertebral bodies (T4–L4) collated from both radiographs and VFA were assessed by each observer using SpineAnalyzer™ and AVERT™.

All VF locations were distributed throughout the thoracic and lumbar spine. The total number and severity of VFs identified through each technique are shown in Table 1. In general, the number and severity of VFs at both subject and vertebral levels varied between the gold standard and the four investigated methods; however, the severity of VF was similar for XR and VFA when using AVERT™. Both methods identified slightly more mild fractures compared with moderate or severe fractures for both observers irrespective of image modality.

**Table 1:** Prevalence (%) of vertebral fractures in study cohort (*n* = 50, 650 vertebrae) at vertebral and subject levels

	DXA AVERT™				XR AVERT™				DXA SpineAnalyzer™				XR SpineAnalyzer™				Reference standard	
	Per vertebra		Per subject		Per vertebra		Per subject		Per vertebra		Per subject		Per vertebra		Per subject		Per vertebra	Per subject
	R1	R2	R1	R2	R1	R2	R1	R2	R1	R2	R1	R2	R1	R2	R1	R2		
No fracture	554 (85%)	502 (77%)	18 (36%)	10 (20%)	561 (86%)	557 (85%)	18 (64%)	19 (38%)	558 (86%)	611 (94%)	17 (34%)	30 (60%)	597 (92%)	616 (95%)	30 (60%)	37 (74%)	475 (73%)	16 (32%)
At least one mild fracture (≤ 24% loss of height)	59 (9%)	85 (13%)	28 (56%)	37 (74%)	48 (7%)	47 (7%)	29 (58%)	27 (54%)	56 (9%)	26 (4%)	31 (62%)	16 (32%)	23 (4%)	17 (3%)	16 (32%)	11 (22%)	132 (20%)	31 (62%)
At least one moderate fracture (25% to 40% loss of height)	22 (3%)	51 (8%)	15 (30%)	26 (52%)	27 (4%)	37 (6%)	17 (34%)	14 (28%)	24 (4%)	12 (2%)	13 (26%)	7 (14%)	16 (2%)	9 (1%)	11 (22%)	6 (12%)	41 (6%)	8 (16%)
At least one severe fracture (≥ 41% loss of height)	15 (2%)	12 (2%)	4 (8%)	5 (10%)	14 (2%)	9 (1%)	4 (8%)	4 (8%)	12 (2%)	1 (˂ 1%)	4 (8%)	1 (2%)	14 (2%)	8 (1%)	3 (6%)	3 (6%)	2 (0.3%)	2 (4%)

**Fig. 3:**
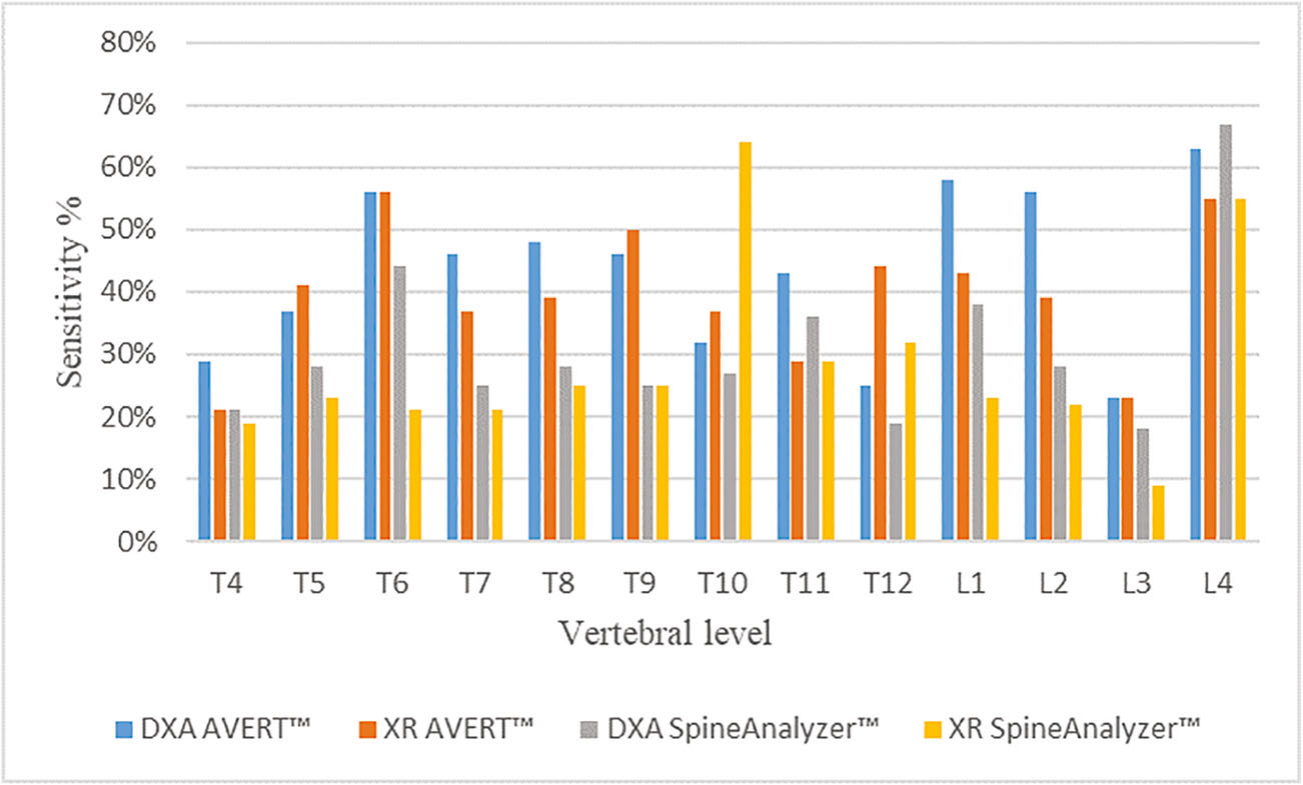
Sensitivity identified for all techniques per vertebral level against the ‘gold standard’ (consensus read by three experienced paediatric radiologists using spine radiographs)

**Fig. 4:**
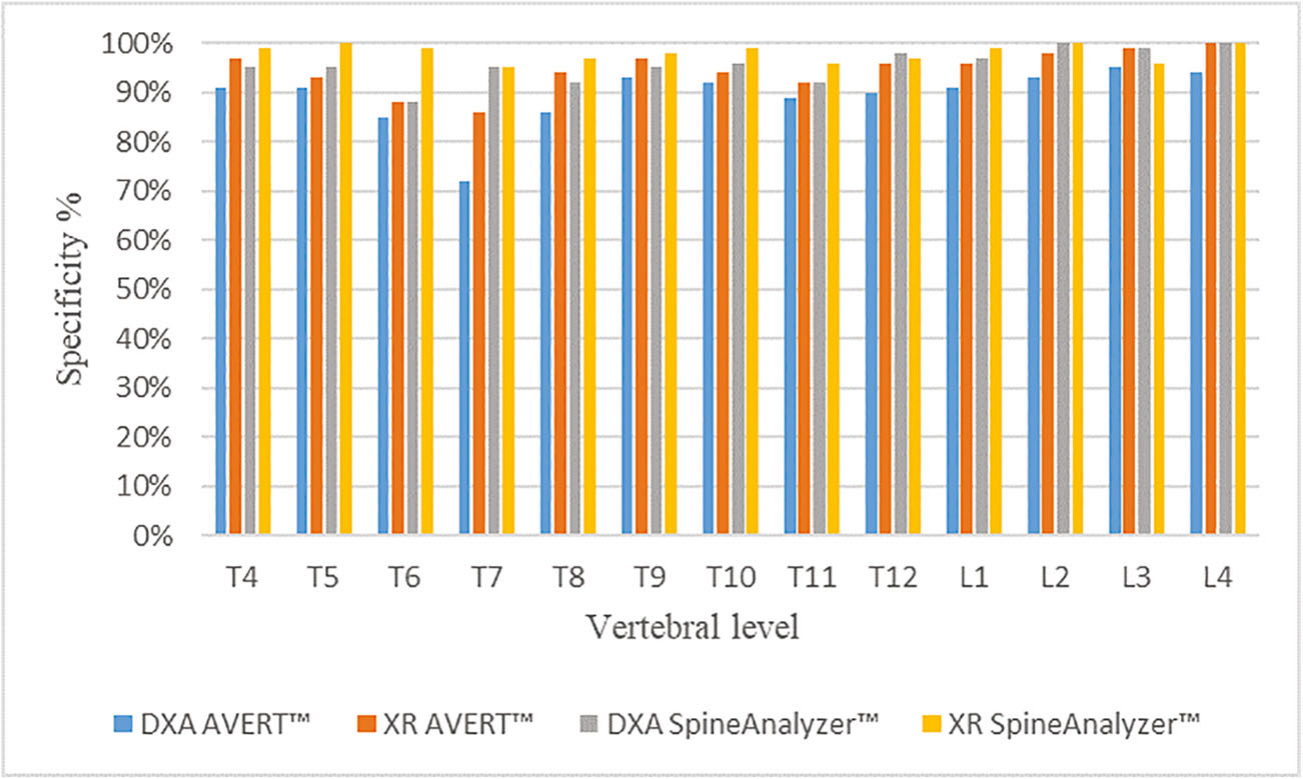
Specificity identified for all techniques per vertebral level against the ‘gold standard’ (consensus read by three experienced paediatric radiologists using spine radiographs)

Sensitivity and specificity of AVERT™ and SpineAnalyzer™ per vertebral level for both modalities (DXA and XR) for all vertebrae from T4 to L4 are shown in Figs. 3 and 4, respectively. Sensitivity, specificity, reliability (kappa, 95%CI) and false-negative and false-positive rates of SpineAnalyzer™ and AVERT™ for both modalities are summarised in Table 2.

**Table 2:** Diagnostic accuracy of AVERT™ and SpineAnalyzer™ for vertebral fracture diagnosis in children

Subject		DXA AVERT™	XR AVERT™	DXA SpineAnalyzer™	XR SpineAnalyzer™
All subjects (50 subjects)	Sensitivity (%)	41	36	31	26
	Specificity (%)	91	95	96	98
	False-negative rate (%)	59	64	69	75
	False-positive rate (%)	9	5	4	2
	Kappa (95%CI)	0.34 (0.26, 0.40)	0.37 (0.27, 0.46)	0.31 (0.21, 0.44)	0.26 (0.16, 0.35)
29 girls	Sensitivity (%)	63	53	57	38
	Specificity (%)	79	85	81	90
	False-negative rate (%)	37	47	43	62
	False-positive rate (%)	21	15	19	10
	Kappa (95%CI)	0.29 (0.19, 0.39)	0.32 (0.21, 0.42)	0.29 (0.20, 0.34)	0.26 (0.11, 0.36)
21 boys	Sensitivity (%)	56	51	57	37
	Specificity (%)	82	82	81	90
	False-negative rate (%)	44	49	43	63
	False-positive rate (%)	18	18	19	10
	Kappa (95%CI)	0.31 (0.20, 0.42)	0.32 (0.19, 0.45)	0.29 (0.19, 0.29)	0.24 (0.13, 0.27)
5–10 years (23 subjects)	Sensitivity (%)	57	53	55	33
	Specificity (%)	88	79	77	90
	False-negative rate (%)	43	47	45	67
	False-positive rate (%)	12	21	23	10
	Kappa (95%CI)	0.36 (0.22, 0.44)	0.33 (0.22, 0.45)	0.30 (0.20, 0.42)	0.22 (0.13, 0.33)
≥ 10–15 years (27 subjects)	Sensitivity (%)	52	46	42	35
	Specificity (%)	88	84	79	91
	False-negative rate (%)	48	54	58	65
	False-positive rate (%)	12	16	21	9
	Kappa (95%CI)	0.33 (0.21, 0.42)	0.30 (0.19, 0.44)	0.25 (0.15, 0.35)	0.19 (0.08, 0.25)

**Fig. 5:**
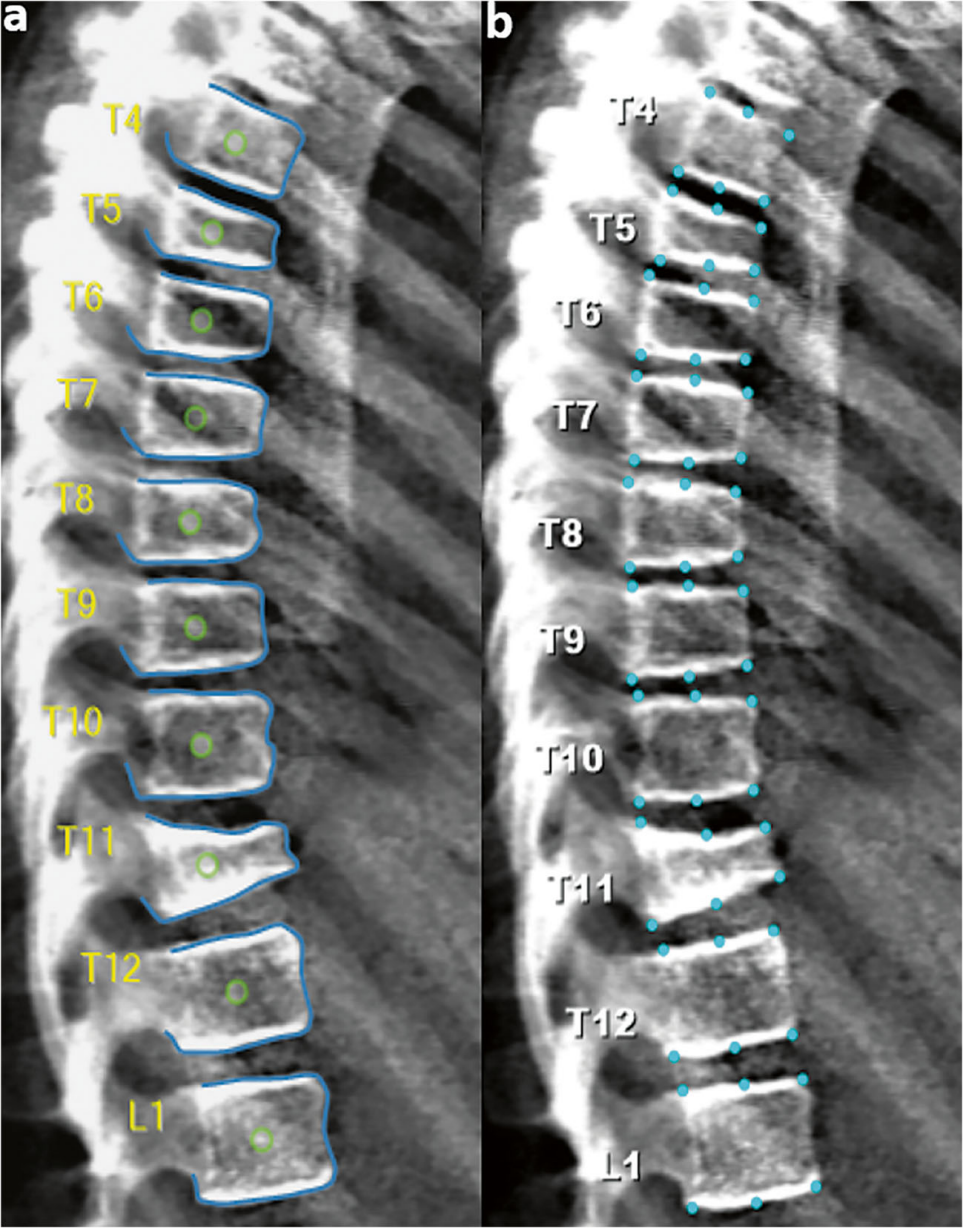
A 10-year-old child with osteogenesis imperfecta. Lateral spine DXA image analysed by AVERT™ (**a**) and SpineAnalyzer™ (**b**) which illustrates the following: Agreement: both programs identified a severe fracture at T11, moderate fractures at T5 and T6, mild fractures at T7 and T8; disagreement: T9 identified as mild fracture by AVERT™ but normal by SpineAnalyzer™; gold standard values: T5, T7, T8 and T9 classified as mild fractures, T6 as normal and T11 as a moderate fracture

Figure 5 shows the agreement between the two programs for DXA images. Overall, there was fair agreement (assessed by kappa statistics) between the four techniques and the consensus evaluation in terms of identifying VF: the average kappa score ranged from 0.26 to 0.37 (95%CI 0.16, 0.46), with XR SpineAnalyzer™ having the lowest score 0.26 (95%CI 0.26, 0.35) and XR AVERT™ having the highest score of 0.37 (95%CI 0.27, 0.46). However, no statistically significant differences were noticed between all the techniques assessed.

Table 3 summarises inter- and intra-observer agreement of all four methods for the two observers. There was a moderate inter-observer agreement between the observers for all methods, with kappa ranging from 0.41 to 0.47 (95%CI 0.25–0.66). In contrast, intra-observer agreement ranged from moderate to good, with mean kappa values for R1 and R2 ranging from 0.50 to 0.79 and 0.59 to 0.78, respectively; SpineAnalyzer™ XR had the lowest score for both observers. For AVERT™, kappa scores for R1 and R2 using VFA were 0.79 (95%CI 0.69, 0.90) and 0.73 (95%CI 0.66, 0.82), respectively.

**Table 3:** Summary of inter and intra-observer agreement for all methods

	Method		Observer	Kappa		
	Software	Modality		Mean	Min	Max
Inter-observer Agreement	AVERT™	DXA	R1 vs R2	0.47	0.27	0.66
	AVERT™	Radiographs	R1 vs R2	0.46	0.21	0.77
	SpineAnalyzer™	DXA	R1 vs R2	0.41	0.25	0.65
	SpineAnalyzer™	Radiographs	R1 vs R2	0.42	0.14	0.73
Intra-observer Agreement	AVERT™	DXA	R1	0.79	0.57	1.00
			R2	0.73	0.41	1.00
		Radiographs	R1	0.78	0.57	1.00
			R2	0.77	0.34	1.00
	SpineAnalyzer™	DXA	R1	0.66	0.34	1.00
			R2	0.78	0.54	1.00
		Radiographs	R1	0.50	0.30	0.69
			R2	0.59	0.41	1.00

Table 4 summarises the overall results of this current study and compares with those of all previous studies that have evaluated semi-automated software techniques in children [7, 8, 12, 15].

**Table 4:** Summary of diagnostic accuracy and observer agreement results of semi-automated software techniques in children

Study	Gold standard (radiographs)	Method		Sensitivity (%)	Specificity (%)	False-negative rate (%)	False-positive rate (%)	Inter-observer agreement (kappa)	Intra-observer agreement (kappa)
		Software	Modality						
A Kyriakou et al [7]	Non-radiologist reader	Six-point analysis	DXA	75	98	–	–	0.79	–
Crabtree et al [8]	An expert paediatric radiologist	Six-point analysis	DXA	79	71	3	25	0.32	–
Alqahtani et al [12]	Consensus arrived by three paediatric radiologists	Six-point analysis (SpineAnalyzer™)	Radiographs	18	97	–	–	0.05–0.47	0.25–0.61
Diacinti et al [15]	Consensus of two skeletal radiologists	Six-point analysis (the Hologic QDR Physician’s Viewer software) (version 7.02)	DXA	66	95	59	10	0.71	–
Current study	Consensus arrived by three paediatric radiologists	Six-point analysis (SpineAnalyzer™)	DXA	31	96	69	4	0.41	0.73–0.79
			Radiographs	26	98	75	2	0.42	0.50–0.59
Current study	Consensus arrived by three paediatric radiologists	33-point analysis (AVERT™)	DXA	41	91	59	9	0.47	0.73–0.79
			Radiographs	36	95	64	5	0.46	0.77–0.78

The time taken by R1 and R2 per image/patient averaged 8 ± 3.45 min (range, 6–14) and 6 ± 2.01 min (range, 4–9 min) respectively for AVERT™ and 6 ± 2.14 min (range, 3–10) and 3 ± 1.14 min (range 2–7 min) respectively for SpineAnalyzer™.

## Discussion

According to the ISCD criteria, the definition of osteoporosis in children is dependent on the identification of one or more VFs. In the absence of VFs, the diagnosis may be made depending on the presence of a bone mineral density *Z*-score of ≤ − 2.0, as well as the number of long bone fractures sustained by the ages of 10 (≥ 2) and 19 (≥ 3) years [1]. It is therefore important to diagnose VF in children at an early stage to allow appropriate treatment plans to be established, such as bisphosphonates, which treat existing fractures as well as reduce the risk of future fractures [16].

Although there are several commercially available programs for quantitative vertebral morphometry assessment in adults, there is as yet no specific semi-automated software for children. In adult subjects, the agreement between observers using 6-point technique programs, e.g. SpineAnalyzer™ (Optasia Medical) and MorphoXpress (MorphoXpress, P&G Pharmaceuticals), has been reported to be higher than that in this study [17–21]. These previous studies show that the 6-point technique programs have very high sensitivity and specificity, reaching 98% and 99%, respectively, and excellent inter-observer agreement of 99%, with kappa ranging from 0.86 to 0.97. In fact, these adult studies show significantly higher diagnostic accuracy than those of all previous studies evaluating 6-point semi-automated programs in children [7, 8, 12, 16].

The purpose of this current study therefore was to ascertain whether observer reliability and diagnostic accuracy of MXA for the identification of VF in children would be improved by using a 33-point semi-automated program compared with the 6-point program for either VFA or radiographs. We used images from 50 subjects used for a previous study [12]. To our knowledge, this is the first report to assess two programs on two different modalities (VFA and radiographs) for the identification of VF in children.

Compared with the consensus reached by the three radiology experts, the overall sensitivity of the 6- and 33-point semi-automated techniques ranged from 26 to 31% and 36 to 41%, respectively. These results are slightly higher than the results from a previous study, in which five readers with different levels of experience assessed the same version of the SpineAnalyzer™ software on 137 radiographs and showed overall sensitivity of only 18% (95%CI 14–2), while overall specificity was 97% (95%CI 97–98) [12]. The 50 images used in the current study were randomly selected from the 137 used in [12] and showed improved overall sensitivity and specificity for SpineAnalyzer™ of 26% to 31% and 96% to 98% respectively and 36% to 41% and 91% to 95% respectively for AVERT™.

In the current study, validity parameters for both software programs were somewhat comparable with those of previous studies [7, 8, 15] (Table 4). For example, sensitivity and specificity for the other three studies ranged from 66 to 79% and 71 to 98%, respectively. The current study has the strength of using a consensus read by three paediatric radiologists, each with a minimum of 13-year experience, as the reference standard.

We have demonstrated that MXA on DXA images is comparable with the MXA on radiographs for identifying clinically significant osteoporotic fractures irrespective of the software program. However, MXA has low diagnostic accuracy and poor observer reliability, with high false-negative rate. Both programs underdiagnosed the prevalence of mild fractures; of the 132 reference standard mild vertebral fractures, only 59, 48, 56 and 23 were identified by DXA AVERT™, XR AVERT™, DXA SpineAnalyzer™ and XR SpineAnalyzer™ by R1 respectively and 85, 47, 26 and 17 by R2, respectively. Moderate and severe vertebral fractures (≥ 25% loss of height in the vertebral body) are readily identified by the naked eye, it is the detection of mild fractures that is clinically problematic [8]. Far from improving the detection of mild fractures, it would seem that MXA underdiagnosed them. The inability to differentiate normal physiological wedging from fracture may account for the low diagnostic accuracy of MXA. We are not aware of any peer-reviewed studies which have comparative data on the normal age- and sex-related values of individual vertebral levels in children. However, a recent study by Jaremko et al in 404 children on glucocorticoid treatment summarises normal variants at different ages and stages of development that may mimic fracture [22].

Despite the limitation of the increased reading time associated with AVERT™, it showed slightly higher accuracy for the diagnosis of VF in children compared with SpineAnalyzer™. However, for both programs, the time was longer in subjects with moderate and/or severe VFs compared with those with no fracture.

Although studies have shown the utility of the biplanar EOS system, e.g. it has been shown to reliably assess spinal and pelvic alignment in the sagittal plane [23], we are not aware of any study that has compared it with radiographs and/or DXA for the diagnosis of vertebral fractures in children. EOS has the advantages of high image quality, low radiation dose and rapid acquisition time. The only disadvantage would be that patients would still require a DXA scan for bone density assessment. Nevertheless, further research studies are worthwhile in order to assess the diagnostic accuracy of vertebral fracture in children using EOS.

The poor observer reliability for both programs may have some explanations. First, there is an inherent subjectivity related to the semi-automated placement of points. Since the placement of these points still relies heavily on the experience of the observer, the correct location of the points can be problematic. Secondly, both programs use the Genant system as their reference, which bases the assessment only on the loss of height of vertebral bodies, while the gold standard uses the sABQ method, which is a visual method that takes account of alterations in the vertebral endplates which may be non-fracture related. Currently, the authors believe that visual methods such as the sABQ approach are more accurate methods of assessing VFs in children.

## Conclusion

Our results show that AVERT™ has a slightly higher accuracy for diagnosis of VF in children compared with SpineAnalyzer™, but both methods have low diagnostic accuracy and observer reliability and we conclude that until the software programs have been specifically improved, or new software developed, MXA cannot be used as a diagnostic tool for VF diagnosis in children.

## Abbreviations

BMD: Bone mineral density
CI: Confidence interval
DXA: Dual-energy X-ray absorptiometry
ISCD: International Society for Clinical Densitometry
MSK: Musculoskeletal
MXA: Morphometric analysis
sABQ: Simplified algorithm-based qualitative
VF: Vertebral fracture
VFA: Vertebral fracture assessment
XR: Radiograph
